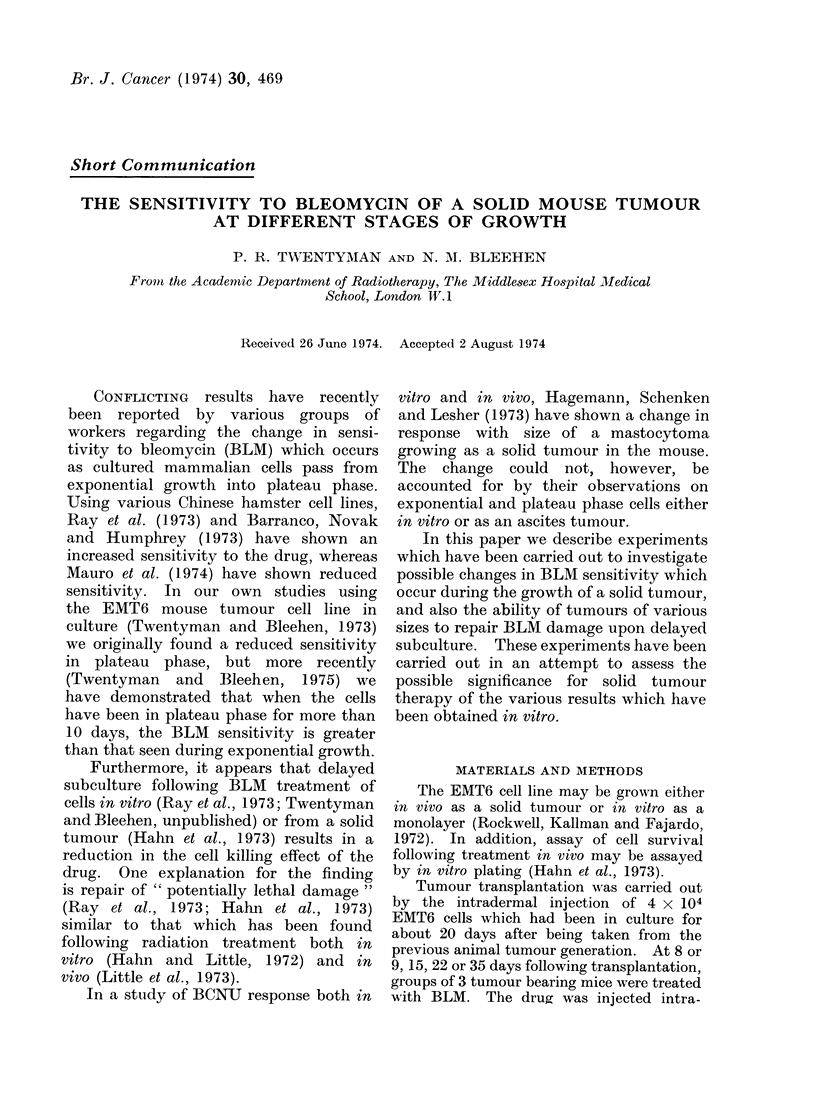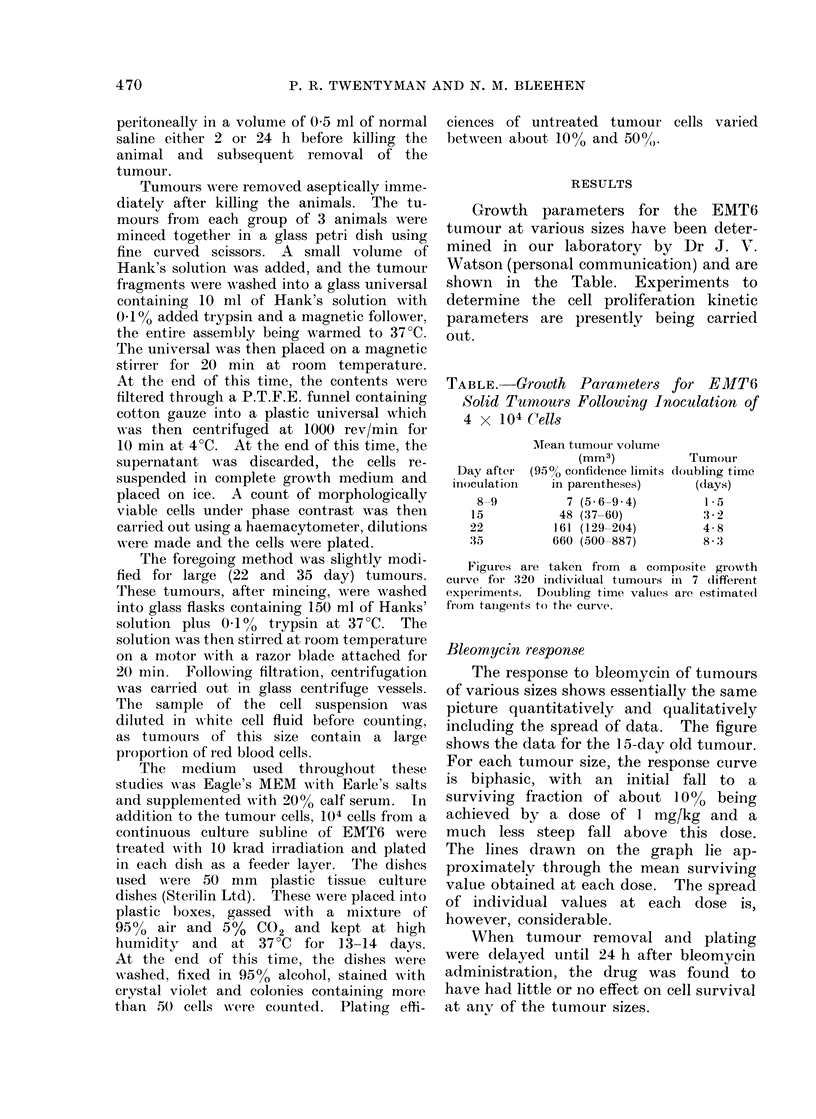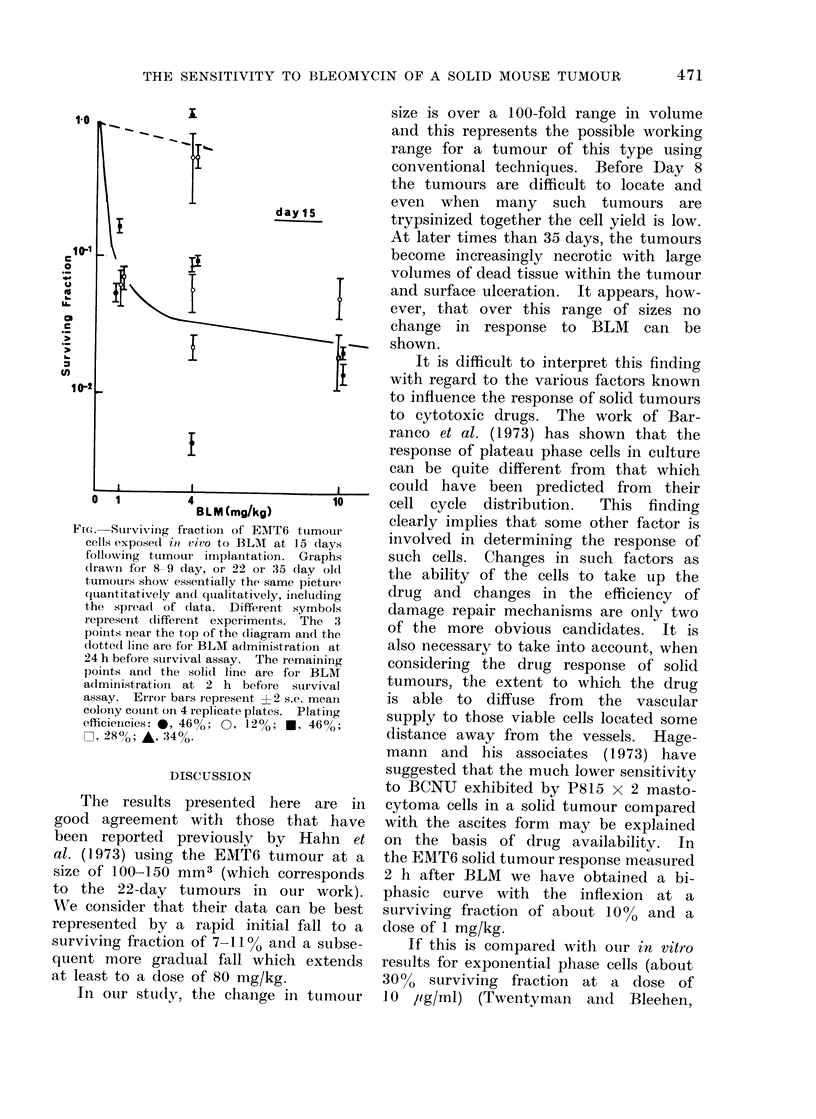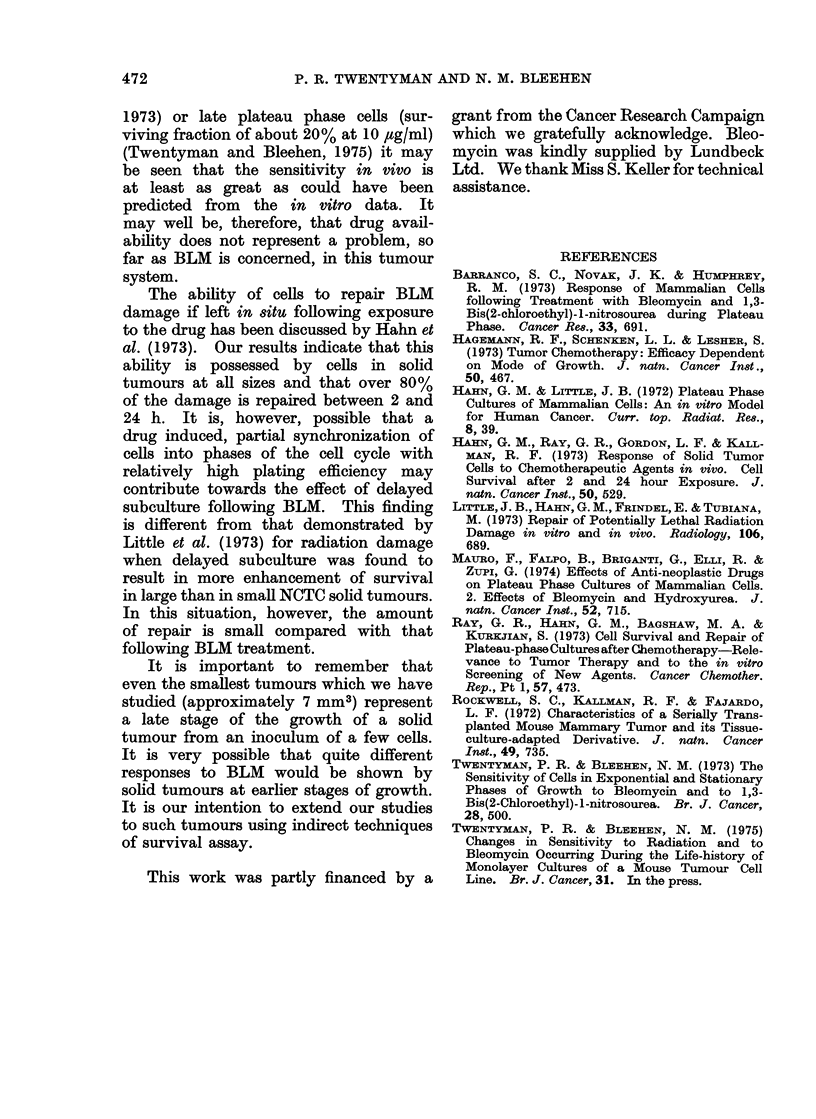# The sensitivity to bleomycin of a solid mouse tumour at different stages of growth.

**DOI:** 10.1038/bjc.1974.222

**Published:** 1974-11

**Authors:** P. R. Twentyman, N. M. Bleehen


*Br. J. Cancer* (1974) **30**, 469

***Short Communication***

### THE SENSITIVITY TO BLEOMYCIN OF A SOLID MOUSE TUMOUR AT DIFFERENT STAGES OF GROWTH

P. R. TWENTYMAN AND N. M. BLEEHEN

*From the Academic Department of Radiotherapy, The Middlesex Hospital Medical School, London W.1*

Received 26 June 1974. Accepted 2 August 1974

CONFLICTING results have recently been reported by various groups of workers regarding the change in sensitivity to bleomycin (BLM) which occurs as cultured mammalian cells pass from exponential growth into plateau phase. Using various Chinese hamster cell lines, Ray *et al.* (1973) and Barranco, Novak and Humphrey (1973) have shown an increased sensitivity to the drug, whereas Mauro *et al.* (1974) have shown reduced sensitivity. In our own studies using the EMT6 mouse tumour cell line in culture (Twentyman and Bleehen, 1973) we originally found a reduced sensitivity in plateau phase, but more recently (Twentyman and Bleehen, 1975) we have demonstrated that when the cells have been in plateau phase for more than 10 days, the BLM sensitivity is greater than that seen during exponential growth.

Furthermore, it appears that delayed subculture following BLM treatment of cells *in vitro* (Ray *et al.*, 1973; Twentyman and Bleehen, unpublished) or from a solid tumour (Hahn *et al.*, 1973) results in a reduction in the cell killing effect of the drug. One explanation for the finding is repair of " potentially lethal damage " (Ray *et al.*, 1973; Hahn *et al.*, 1973) similar to that which has been found following radiation treatment both *in vitro* (Hahn and Little, 1972) and *in vivo* (Little *et al.*, 1973).

In a study of BCNU response both *in vitro* and *in vivo*, Hagemann, Schenken and Lesher (1973) have shown a change in response with size of a mastocytoma growing as a solid tumour in the mouse. The change could not, however, be accounted for by their observations on exponential and plateau phase cells either *in vitro* or as an ascites tumour.

In this paper we describe experiments which have been carried out to investigate possible changes in BLM sensitivity which occur during the growth of a solid tumour, and also the ability of tumours of various sizes to repair BLM damage upon delayed subculture. These experiments have been carried out in an attempt to assess the possible significance for solid tumour therapy of the various results which have been obtained *in vitro*.

### MATERIALS AND METHODS

The EMT6 cell line may be grown either *in vivo* as a solid tumour or *in vitro* as a monolayer (Rockwell, Kallman and Fajardo, 1972). In addition, assay of cell survival following treatment *in vivo* may be assayed by *in vitro* plating (Hahn *et al.*, 1973).

Tumour transplantation was carried out by the intradermal injection of $4 \times 10^4$ EMT6 cells which had been in culture for about 20 days after being taken from the previous animal tumour generation. At 8 or 9, 15, 22 or 35 days following transplantation, groups of 3 tumour bearing mice were treated with BLM. The drug was injected intraperitoneally in a volume of 0·5 ml of normal saline either 2 or 24 h before killing the animal and subsequent removal of the tumour.

Tumours were removed aseptically immediately after killing the animals. The tumours from each group of 3 animals were minced together in a glass petri dish using fine curved scissors. A small volume of Hank's solution was added, and the tumour fragments were washed into a glass universal containing 10 ml of Hank's solution with 0·1% added trypsin and a magnetic follower, the entire assembly being warmed to 37°C. The universal was then placed on a magnetic stirrer for 20 min at room temperature. At the end of this time, the contents were filtered through a P.T.F.E. funnel containing cotton gauze into a plastic universal which was then centrifuged at 1000 rev/min for 10 min at 4°C. At the end of this time, the supernatant was discarded, the cells resuspended in complete growth medium and placed on ice. A count of morphologically viable cells under phase contrast was then carried out using a haemacytometer, dilutions were made and the cells were plated.

The foregoing method was slightly modified for large (22 and 35 day) tumours. These tumours, after mincing, were washed into glass flasks containing 150 ml of Hanks' solution plus 0·1% trypsin at 37°C. The solution was then stirred at room temperature on a motor with a razor blade attached for 20 min. Following filtration, centrifugation was carried out in glass centrifuge vessels. The sample of the cell suspension was diluted in white cell fluid before counting, as tumours of this size contain a large proportion of red blood cells.

The medium used throughout these studies was Eagle's MEM with Earle's salts and supplemented with 20% calf serum. In addition to the tumour cells, $10^4$ cells from a continuous culture subline of EMT6 were treated with 10 krad irradiation and plated in each dish as a feeder layer. The dishes used were 50 mm plastic tissue culture dishes (Sterilin Ltd). These were placed into plastic boxes, gassed with a mixture of 95% air and 5% $CO_2$ and kept at high humidity and at 37°C for 13–14 days. At the end of this time, the dishes were washed, fixed in 95% alcohol, stained with crystal violet and colonies containing more than 50 cells were counted. Plating efficiences of untreated tumour cells varied between about 10% and 50%.

### RESULTS

Growth parameters for the EMT6 tumour at various sizes have been determined in our laboratory by Dr J. V. Watson (personal communication) and are shown in the Table. Experiments to determine the cell proliferation kinetic parameters are presently being carried out.

TABLE.—*Growth Parameters for EMT6 Solid Tumours Following Inoculation of $4 \times 10^4$ Cells*

| Day after inoculation | Mean tumour volume ($mm^3$) (95% confidence limits in parentheses) | Tumour doubling time (days) |
|---|---|---|
| 8–9 | 7 (5·6–9·4) | 1·5 |
| 15 | 48 (37–60) | 3·2 |
| 22 | 161 (129–204) | 4·8 |
| 35 | 660 (500–887) | 8·3 |

Figures are taken from a composite growth curve for 320 individual tumours in 7 different experiments. Doubling time values are estimated from tangents to the curve.

### *Bleomycin response*

The response to bleomycin of tumours of various sizes shows essentially the same picture quantitatively and qualitatively including the spread of data. The figure shows the data for the 15-day old tumour. For each tumour size, the response curve is biphasic, with an initial fall to a surviving fraction of about 10% being achieved by a dose of 1 mg/kg and a much less steep fall above this dose. The lines drawn on the graph lie approximately through the mean surviving value obtained at each dose. The spread of individual values at each dose is, however, considerable.

When tumour removal and plating were delayed until 24 h after bleomycin administration, the drug was found to have had little or no effect on cell survival at any of the tumour sizes.

FIG.—Surviving fraction of EMT6 tumour cells exposed *in vivo* to BLM at 15 days following tumour implantation. Graphs drawn for 8–9 day, or 22 or 35 day old tumours show essentially the same picture quantitatively and qualitatively, including the spread of data. Different symbols represent different experiments. The 3 points near the top of the diagram and the dotted line are for BLM administration at 24 h before survival assay. The remaining points and the solid line are for BLM administration at 2 h before survival assay. Error bars represent $\pm 2$ s.e. mean colony count on 4 replicate plates. Plating efficiencies: ●, 46%; ○, 12%; ■, 46%; □, 28%; ▲, 34%.

### DISCUSSION

The results presented here are in good agreement with those that have been reported previously by Hahn *et al.* (1973) using the EMT6 tumour at a size of 100–150 mm$^3$ (which corresponds to the 22-day tumours in our work). We consider that their data can be best represented by a rapid initial fall to a surviving fraction of 7–11% and a subsequent more gradual fall which extends at least to a dose of 80 mg/kg.

In our study, the change in tumour size is over a 100-fold range in volume and this represents the possible working range for a tumour of this type using conventional techniques. Before Day 8 the tumours are difficult to locate and even when many such tumours are trypsinized together the cell yield is low. At later times than 35 days, the tumours become increasingly necrotic with large volumes of dead tissue within the tumour and surface ulceration. It appears, however, that over this range of sizes no change in response to BLM can be shown.

It is difficult to interpret this finding with regard to the various factors known to influence the response of solid tumours to cytotoxic drugs. The work of Barranco *et al.* (1973) has shown that the response of plateau phase cells in culture can be quite different from that which could have been predicted from their cell cycle distribution. This finding clearly implies that some other factor is involved in determining the response of such cells. Changes in such factors as the ability of the cells to take up the drug and changes in the efficiency of damage repair mechanisms are only two of the more obvious candidates. It is also necessary to take into account, when considering the drug response of solid tumours, the extent to which the drug is able to diffuse from the vascular supply to those viable cells located some distance away from the vessels. Hagemann and his associates (1973) have suggested that the much lower sensitivity to BCNU exhibited by P815 $\times$ 2 mastocytoma cells in a solid tumour compared with the ascites form may be explained on the basis of drug availability. In the EMT6 solid tumour response measured 2 h after BLM we have obtained a biphasic curve with the inflexion at a surviving fraction of about 10% and a dose of 1 mg/kg.

If this is compared with our *in vitro* results for exponential phase cells (about 30% surviving fraction at a dose of 10 $\mu$g/ml) (Twentyman and Bleehen,

1973) or late plateau phase cells (surviving fraction of about 20% at 10 $\mu$g/ml) (Twentyman and Bleehen, 1975) it may be seen that the sensitivity *in vivo* is at least as great as could have been predicted from the *in vitro* data. It may well be, therefore, that drug availability does not represent a problem, so far as BLM is concerned, in this tumour system.

The ability of cells to repair BLM damage if left *in situ* following exposure to the drug has been discussed by Hahn *et al.* (1973). Our results indicate that this ability is possessed by cells in solid tumours at all sizes and that over 80% of the damage is repaired between 2 and 24 h. It is, however, possible that a drug induced, partial synchronization of cells into phases of the cell cycle with relatively high plating efficiency may contribute towards the effect of delayed subculture following BLM. This finding is different from that demonstrated by Little *et al.* (1973) for radiation damage when delayed subculture was found to result in more enhancement of survival in large than in small NCTC solid tumours. In this situation, however, the amount of repair is small compared with that following BLM treatment.

It is important to remember that even the smallest tumours which we have studied (approximately 7 mm$^3$) represent a late stage of the growth of a solid tumour from an inoculum of a few cells. It is very possible that quite different responses to BLM would be shown by solid tumours at earlier stages of growth. It is our intention to extend our studies to such tumours using indirect techniques of survival assay.

This work was partly financed by a grant from the Cancer Research Campaign which we gratefully acknowledge. Bleomycin was kindly supplied by Lundbeck Ltd. We thank Miss S. Keller for technical assistance.

### REFERENCES

Barranco, S. C., Novak, J. K. & Humphrey, R. M. (1973) Response of Mammalian Cells following Treatment with Bleomycin and 1,3-Bis(2-chloroethyl)-1-nitrosourea during Plateau Phase. *Cancer Res.*, **33**, 691.

Hagemann, R. F., Schenken, L. L. & Lesher, S. (1973) Tumor Chemotherapy: Efficacy Dependent on Mode of Growth. *J. natn. Cancer Inst.*, **50**, 467.

Hahn, G. M. & Little, J. B. (1972) Plateau Phase Cultures of Mammalian Cells: An *in vitro* Model for Human Cancer. *Curr. top. Radiat. Res.*, **8**, 39.

Hahn, G. M., Ray, G. R., Gordon, L. F. & Kallman, R. F. (1973) Response of Solid Tumor Cells to Chemotherapeutic Agents *in vivo*. Cell Survival after 2 and 24 hour Exposure. *J. natn. Cancer Inst.*, **50**, 529.

Little, J. B., Hahn, G. M., Frindel, E. & Tubiana, M. (1973) Repair of Potentially Lethal Radiation Damage *in vitro* and *in vivo*. *Radiology*, **106**, 689.

Mauro, F., Falpo, B., Briganti, G., Elli, R. & Zupi, G. (1974) Effects of Anti-neoplastic Drugs on Plateau Phase Cultures of Mammalian Cells. 2. Effects of Bleomycin and Hydroxyurea. *J. natn. Cancer Inst.*, **52**, 715.

Ray, G. R., Hahn, G. M., Bagshaw, M. A. & Kurkjian, S. (1973) Cell Survival and Repair of Plateau-phase Cultures after Chemotherapy—Relevance to Tumor Therapy and to the *in vitro* Screening of New Agents. *Cancer Chemother. Rep.*, Pt 1, **57**, 473.

Rockwell, S. C., Kallman, R. F. & Fajardo, L. F. (1972) Characteristics of a Serially Transplanted Mouse Mammary Tumor and its Tissue-culture-adapted Derivative. *J. natn. Cancer Inst.*, **49**, 735.

Twentyman, P. R. & Bleehen, N. M. (1973) The Sensitivity of Cells in Exponential and Stationary Phases of Growth to Bleomycin and to 1,3-Bis(2-Chloroethyl)-1-nitrosourea. *Br. J. Cancer*, **28**, 500.

Twentyman, P. R. & Bleehen, N. M. (1975) Changes in Sensitivity to Radiation and to Bleomycin Occurring During the Life-history of Monolayer Cultures of a Mouse Tumour Cell Line. *Br. J. Cancer*, **31**. In the press.